# Transforming Cardiovascular Risk Prediction: A Review of Machine Learning and Artificial Intelligence Innovations

**DOI:** 10.3390/life15010094

**Published:** 2025-01-14

**Authors:** Dimitrios-Ioannis Kasartzian, Thomas Tsiampalis

**Affiliations:** Department of Nutrition and Dietetics, School of Physical Education, Sports and Dietetics, University of Thessaly, 42132 Trikala, Greece; kasartziandimitris@gmail.com

**Keywords:** cardiovascular disease, risk prediction, machine learning, artificial intelligence, deep learning, precision medicine

## Abstract

Cardiovascular diseases (CVDs) remain a leading cause of global mortality and morbidity. Traditional risk prediction models, while foundational, often fail to capture the multifaceted nature of risk factors or leverage the expanding pool of healthcare data. Machine learning (ML) and artificial intelligence (AI) approaches represent a paradigm shift in risk prediction, offering dynamic, scalable solutions that integrate diverse data types. This review examines advancements in AI/ML for CVD risk prediction, analyzing their strengths, limitations, and the challenges associated with their clinical integration. Recommendations for standardization, validation, and future research directions are provided to unlock the potential of these technologies in transforming precision cardiovascular medicine.

## 1. Introduction

Cardiovascular diseases (CVDs) continue to represent a major global health challenge, contributing significantly to morbidity and mortality worldwide, despite advances in prevention and early disease intervention [1]. Traditional risk prediction models, like the Framingham Risk Score (FRS) [2] and the Systematic Coronary Risk Evaluation (SCORE) model [3], have long been used to estimate an individual’s risk of developing CVD. These models rely heavily on classical statistical regression approaches to assess common risk factors, like age, sex, blood pressure, and lipid profiles. However, such methods, while effective to an extent, come with limitations. They tend to oversimplify complex, nonlinear relationships between risk factors and outcomes and may lack the flexibility to incorporate evolving patient data and more advanced biomarkers. In addition, these models are often region-specific, leading to challenges in generalizability across diverse populations. Moreover, traditional models are prone to underestimating risk in younger individuals [4] or certain demographic groups, such as women [5] and racial minorities [6], due to a lack of personalized or population-specific predictors.

In recent years, the increasing complexity of healthcare data coupled with the rapid digitalization of medical records have exposed the inadequacies of traditional CVD risk models. These models often rely on static and linear relationships between a limited number of risk factors, making them insufficient for capturing the intricate and multifactorial nature of CVDs [7]. To address these challenges, machine learning (ML) and artificial intelligence (AI) have emerged as promising tools in risk prediction. More precisely, ML, with its ability to process large, multidimensional datasets and uncover non-obvious patterns, offers a dynamic and scalable alternative to conventional statistical methods [8].

A key advantage of ML is its capacity to manage diverse and complex data types—ranging from electronic health records (EHRs) to imaging, genetic, and lifestyle information—which allows for more holistic and individualized risk assessments. In particular, deep learning (DL), a specialized subset of ML, employs neural networks that simulate the human brain’s structure and function. These networks are designed to analyze and learn from complex medical data in layers, making it possible to detect subtle, nonlinear relationships between variables [9]. This ability to extract features automatically from raw data facilitates personalized and more accurate risk assessments, particularly when applied to high-dimensional data such as medical imaging, genomics, and proteomics [10]. Moreover, natural language processing (NLP), another branch of AI, can mine unstructured data from medical records, such as physician notes, clinical reports, and patient histories, to extract meaningful insights and further enhance the predictive power of ML models [11]. By integrating these different forms of structured and unstructured data, AI models can move beyond traditional risk factors to include novel predictors, such as genetic polymorphisms [12], proteomic signatures [10], and real-time physiological monitoring data from wearable devices [13].

These advancements are transforming the landscape of cardiovascular risk prediction. For example, AI models have shown promise in integrating imaging data, such as those from coronary computed tomography angiography (CCTA) [14] and cardiac magnetic resonance (CMR) imaging, to identify early signs of subclinical atherosclerosis and plaque vulnerability, providing more precise predictions of future cardiovascular events. Additionally, incorporating genetic information through AI models allows for the identification of polygenic risk scores that can stratify individuals at high genetic risk for CVD [15]. Biomarker profiles, such as levels of inflammatory markers or cardiac-specific proteins like troponins, can also be integrated to improve prediction accuracy [16]. In this evolving context, ML-based CVD risk prediction models are not only improving risk stratification but are also opening the door for real-time, continuous monitoring and prediction [17,18].

As the field moves toward AI-driven models, the potential to improve CVD risk prediction and prevention is immense. The present review article examines the application of ML and AI in CVD risk prediction, emphasizing their potential to transform precision medicine. The present review analyses diverse AI techniques, such as deep learning, ensemble methods, and natural language processing, highlighting their capabilities to integrate and analyze multidimensional datasets, including genomics, proteomics, imaging, and wearable device data. A literature review was conducted using PubMed, with a focus on studies published between 6 April 2019 and 9 August 2024. Search terms included “cardiovascular disease”, “machine learning”, and “risk prediction”, with English-language publications prioritized. The included studies span various methodologies and populations, providing an overview of the strengths, limitations, and clinical implications of AI-driven CVD risk prediction.

## 2. Theoretical Background of Machine Learning and Artificial Intelligence

ML and AI have revolutionized various domains, including healthcare, by enabling the analysis of complex datasets to uncover patterns and make predictions. ML, a subset of AI, involves the use of algorithms that learn from data to improve their performance without explicit programming. It is broadly categorized into supervised learning (using labeled data), unsupervised learning (detecting patterns in unlabeled data), and reinforcement learning (optimizing decisions through trial and error). The application of AI and ML in healthcare began to gain significant attention in the scientific literature around the early 2000s, with a notable increase in publications from the mid-2010s. This surge coincided with advancements in computational power, the proliferation of big data in healthcare, and the development of more sophisticated algorithms such as deep learning. In cardiovascular medicine, interest in AI and ML has grown exponentially over the last decade, particularly as studies have demonstrated their ability to enhance risk prediction, personalize treatment strategies, and analyze complex datasets, such as medical imaging and genomic data. In the context of cardiovascular medicine, ML models, like random forests, support vector machines, and neural networks, are used for predictive analytics, while advanced approaches such as deep learning, particularly convolutional and recurrent neural networks, facilitate the analysis of high-dimensional data, like medical imaging and genomics. Techniques such as ensemble learning combine multiple models to enhance prediction accuracy and robustness. AI complements ML through methodologies like natural language processing (NLP) and computer vision. NLP enables the extraction of insights from unstructured text data (e.g., medical notes), while computer vision analyzes imaging data for diagnostic and predictive purposes. These capabilities make AI integral to modern healthcare, allowing for holistic, patient-specific insights. Despite their transformative potential, ML and AI face challenges, including the need for high-quality, diverse datasets to prevent model bias and the demand for interpretability to foster trust among clinicians. Addressing these challenges is crucial to ensure equitable and reliable applications in clinical settings.

## 3. Advancements in AI/ML for CVD Risk Prediction

### 3.1. Historical Context and Evolution

Traditional CVD risk models laid the groundwork for systematic risk assessment but are constrained by their reliance on linear relationships and limited data types. For instance, models such as the Framingham Risk Score (FRS) primarily focus on static variables, like age, sex, and blood pressure, neglecting the dynamic and multifactorial nature of cardiovascular risks. However, the evolution of computational tools has introduced models capable of handling these complexities. The majority of studies reviewed here were published in the last five years, reflecting the rapid advancements in this field. Notably, 30.8% of the studies emerged from the United States and Europe, with a trend toward larger sample sizes (46.2% of studies included over 10,000 participants). The increasing diversity of study settings—spanning community-based and institutional frameworks—has also broadened the applicability of AI in various healthcare environments. Ensemble learning methods, such as random forest and gradient boosting, have further advanced predictive modeling by combining multiple weak learners to enhance accuracy and robustness. Deep neural networks, including convolutional and recurrent architectures, have enabled the analysis of complex datasets, such as imaging and longitudinal records, offering insights into both static and temporal patterns of cardiovascular risk. Studies such as Lauber et al. (2022) [19] exemplify the use of advanced lipidomic profiling, employing Ridge regression to achieve significant predictive improvements. By integrating 184 lipid species, the study not only outperformed traditional models but also provided insights into early pathological changes through biomarkers like triacylglycerides and diacylglycerides. Hoogeveen et al. (2020) [20] further demonstrated the application of proteomic data, where biomarkers like GDF-15 and IL-6 revealed inflammatory and metabolic pathways crucial for risk assessment. These advancements mark a shift from reliance on clinical factors alone to a more comprehensive inclusion of molecular data.

### 3.2. Innovations in Predictive Modeling

AI and ML techniques have revolutionized CVD risk prediction by facilitating the integration of diverse data modalities. Advanced lipidomic and proteomic profiling, for instance, has been incorporated into ML models, significantly enhancing predictive accuracy. Studies utilizing Ridge regression demonstrated substantial improvements when integrating these biomarkers. Wu et al. (2024) [21] showcased how lipidomic risk scores (LRS) outperformed traditional models like the FRS, achieving an AUC increase from 0.545 to 0.659, with robust external validation. Proteomic data have also been leveraged to capture inflammatory and metabolic pathways relevant to CVD, as demonstrated by Hoogeveen et al. (2020) [20]. By incorporating biomarkers like GDF-15 and IL-6, the study achieved an AUC of 0.754, underscoring the role of advanced molecular profiling in improving short-term risk predictions. Additionally, the integration of polygenic risk scores, as highlighted in studies like Lauber et al. (2022) [19], has further enriched the predictive power of AI models, showcasing their potential to enhance early disease detection. Temporal modeling has also emerged as a critical innovation. Models such as Dynamic-DeepHit employ attention mechanisms to capture longitudinal data, ensuring that predictions remain relevant even as patient conditions evolve. Similarly, sex-specific models have leveraged predictors like systolic blood pressure variability and BMI to address unique risk profiles. These advancements ensure nuanced predictions, especially in high-risk groups often overlooked by conventional methods. Moreover, the incorporation of non-traditional predictors, such as social and environmental factors, has expanded the scope of risk assessment. Factors like socioeconomic status and environmental exposures are increasingly recognized as critical determinants of health. Atehortúa et al. (2023) [22] demonstrated the utility of exposome-based ML models, which integrated these variables to outperform traditional methods in predicting CVD risk across diverse populations. Dong et al. (2024) [23] further emphasized the importance of tailored models, achieving robust calibration and improved discrimination metrics in sex-specific analyses for Chinese populations with type 2 diabetes mellitus.

### 3.3. Application in Diverse Populations

Addressing disparities in healthcare outcomes remains a pressing challenge, and AI models hold promise for mitigating these inequities. Studies have shown that ML algorithms tailored to population-specific characteristics significantly improve predictive performance. For instance, sex-specific ML models, like those developed by Dong et al. (2024) [23], achieved superior discrimination metrics in predicting 10-year CVD risk in women compared to men. This highlights the ability of AI to fill gaps left by traditional models, which often fail to consider gender-specific variations. Similarly, models trained on datasets including racially and ethnically diverse populations have identified unique risk factors, such as genetic polymorphisms and culturally specific health behaviors. However, 69.2% of studies reviewed were conducted in institutional frameworks, emphasizing the need for community-based research to capture broader, real-world data. The work by An et al. (2021) [24] on the DeepRisk model exemplifies the integration of heterogeneous clinical, diagnostic, and demographic data to achieve superior predictive performance (AUC of 0.8375). This attention-based deep learning framework provided interpretable insights, identifying diabetes, hypertension, and hyperlipidemia as key predictors. Such models ensure that predictions remain adaptable to evolving clinical and demographic contexts, providing a template for addressing disparities in CVD outcomes. Efforts to address data variability, such as the standardization of feature selection and validation protocols, are essential for ensuring equitable model performance. Collaborations across institutions and geographical regions are crucial to developing large, representative datasets. Furthermore, leveraging advanced architectures like attention-based models ensures that predictions remain adaptable to the evolving clinical and demographic landscapes of diverse populations.

Table 1, Table 2 and Table 3 provide a small overview of the most recently developed AI and ML methodologies for CVD risk prediction.

## 4. AI and ML in Cardiovascular Risk Prediction: A Paradigm Shift in Precision Medicine

The emergence of ML and AI approaches in CVD risk prediction represents a paradigm shift in how risk is assessed and managed, marking a transition from traditional linear modelling frameworks to data-driven, multidimensional, and highly dynamic methodologies [32,33]. The majority of the studies reviewed herein consistently demonstrate that ML-based models outperform traditional methods in predictive accuracy, as evidenced by higher AUC values, improved calibration, and better NRIs. These metrics reflect the ability of ML models to offer more nuanced and individualized risk assessments, particularly for subpopulations where traditional models often falter, such as intermediate-risk groups and underserved populations [6]. For instance, models such as Dynamic-DeepHit and DeepRisk leverage longitudinal data and attention mechanisms to capture complex temporal patterns, which static, traditional models are unable to address. By doing so, these approaches account for the dynamic nature of patient risk profiles over time, providing real-time, adaptive predictions that align with the evolving clinical context. Similarly, models incorporating lipidomic and proteomic data extend the predictive scope by offering insights into the molecular mechanisms underlying CVD [34]. These approaches enhance our understanding of pathophysiological processes, such as inflammation, lipid metabolism, and vascular remodeling, providing unique predictive value beyond established clinical and genetic risk factors [10]. For example, lipidomic risk scores have shown significant potential in identifying high-risk individuals, even in cohorts where traditional models, such as the FRS, underperform [35]. The integration of these novel biomarkers underscores the role of ML and AI in bridging the gap between molecular research and clinical application [36].

## 5. Challenges at Development, Validation, and Impact Stages

Despite these advancements, several challenges persist across the translational pathway, encompassing the development, validation, and clinical impact of ML models [37].

### 5.1. Development Stage

Current research on ML applications in CVD risk prediction has been constrained by inadequate sample sizes, geographic and racial disparities, and poor data quality. In many cases, the selection of variables for ML algorithms is performed arbitrarily, raising concerns about model development rigor and interpretability. Ensuring the generalizability of data and reproducibility of methods is critical in epidemiological studies to facilitate clinical application, yet these issues remain inadequately addressed in most studies to date [38]. The consequence is a significant risk of bias, undermining the reliability of findings and their broader applicability. Moreover, the absence of standardized approaches for integrating heterogeneous data, such as imaging, omics, and clinical data, further complicates model development.

### 5.2. Validation Stage

The robustness of ML models relies heavily on internal and external validation. While cross-validation can mitigate some imbalances within datasets, it often fails to capture the full range of real-world clinical scenarios [39]. External validation is pivotal to ensure that ML models perform consistently across diverse populations, yet it remains notably absent in many studies [40]. A frequent challenge is model shrinkage, where predictive performance declines sharply when models are applied to independent external datasets. This limitation poses a significant barrier to clinical adoption, as models that lack validation cannot be confidently deployed in real-world settings.

### 5.3. Impact Stage

The assessment of clinical impact, including effectiveness and cost-effectiveness, remains underexplored. There is a significant gap in evaluating the clinical utility of ML models, particularly in terms of accessibility, interpretability, and outcomes relevant to healthcare practitioners [41]. Moreover, the lack of standardized reporting frameworks further hinders the translation of ML findings into actionable tools [42]. Addressing these gaps requires large-scale studies with access to comprehensive databases, covering various CVD subtypes and employing standardized validation methodologies.

### 5.4. Interpretability and Transparency

Interpretability is a central challenge for the adoption of ML models in clinical practice. While techniques such as SHapley Additive exPlanations (SHAP) and attention mechanisms have improved transparency, many black-box models remain difficult for clinicians to understand and trust [43]. This lack of transparency raises ethical concerns, as predictions that cannot be explained are harder to justify, particularly in cases with critical clinical or legal implications. The adoption of interpretable frameworks and rigorous reporting standards, such as the utilization of the Transparent Reporting of a multivariable prediction model for Individual Prognosis Or Diagnosis (TRIPOD) [44] and the MINimum Information for Medical AI Reporting (MINIMAR) [45], is essential for addressing inconsistencies in data coding and fostering trust in ML systems.

### 5.5. Integration of Diverse Data and Standardization

To enhance the predictive capabilities of ML models, future research should focus on integrating diverse data types, including polygenic risk scores and lipidomic, proteomic, transcriptomic, and metabolomic datasets. Combining these data with phenotypic features, such as coronary artery imaging, will facilitate a more comprehensive understanding of individual CVD phenotypes. Additionally, challenges related to incomplete and inconsistent coding in medical databases, such as those involving ICD terminologies, must be addressed through standardized approaches for data normalization, employing, for example, the Unified Medical Language System (UMLS) [46]. Standardized methodologies will ensure that ML models are robust, reproducible, and clinically relevant. One of the key challenges in the application of ML in CVD risk prediction is the lack of transparency in algorithmic methodologies and the reporting of features used in the studies. As seen in Table 1 and Table 2, many studies do not provide detailed descriptions of the ML algorithms employed or the specific features selected for model development. This lack of transparency poses significant barriers to the reproducibility and validation of these models, limiting their clinical and research utility. To address these challenges, standardized reporting frameworks are essential. Initiatives such as TRIPOD (Transparent Reporting of a multivariable prediction model for Individual Prognosis Or Diagnosis) and MINIMAR (MINimum Information for Medical AI Reporting) provide clear guidelines for reporting prediction model studies, including details on feature selection, data preprocessing, and model development. Adopting such frameworks ensures consistency and completeness in reporting, thereby enhancing the interpretability and reliability of ML models. Furthermore, method development in ML research should prioritize explainability and user-friendly tools that enable clinicians to understand the rationale behind predictions. Explainable AI (XAI) techniques, for instance, can provide insights into the importance of features and the decision-making process of ML algorithms. By fostering transparency, these approaches can help bridge the gap between computational advancements and practical clinical applications. Future studies must also address the heterogeneity in feature selection methods and provide comprehensive documentation of the data and algorithms used. This effort will not only facilitate external validation but also encourage the development of generalizable and equitable models that can be reliably implemented across diverse populations and settings.

### 5.6. Addressing Censoring and Data Limitations

Another critical challenge in long-term CVD risk prediction is censoring, where patients are lost to follow-up due to withdrawal, death, or incomplete data capture. If improperly handled, censoring can bias model performance estimates. While traditional survival analysis models, such as QRESEARCH (cardiovascular risk algorithm) estimated version 3 (QRISK3 score), are equipped to address censoring, ML models like random survival forest offer a promising solution for incorporating time-to-event data [47]. Ensuring the inclusion of survival outcomes in ML models will enhance their clinical relevance, particularly in longitudinal studies.

### 5.7. Influence of Sample Size on ML Models

The variation in sample sizes across studies, as highlighted in Table 3, plays a crucial role in the development, validation, and testing of machine learning (ML) models for cardiovascular disease (CVD) risk prediction. Larger sample sizes generally provide a more diverse and representative dataset, enabling models to learn complex patterns and relationships while reducing the risk of overfitting. This enhances the generalizability of the model to new, unseen data. Conversely, studies with smaller sample sizes face challenges, such as limited representativeness, increased model variance, and overfitting, where the model performs well on the training dataset but poorly on external or independent datasets. Small sample sizes may also exacerbate biases, especially when the dataset is imbalanced with respect to critical variables, such as gender, ethnicity, or age. To address these challenges, researchers can employ strategies such as the following:

Data Augmentation: Generating synthetic data to increase the effective sample size while preserving the characteristics of the original dataset.

Transfer Learning: Leveraging pre-trained models from larger datasets to improve performance on smaller datasets.

External Validation: Validating models on independent datasets from diverse populations to ensure robustness and generalizability.

Additionally, future research should prioritize the development of collaborative, multi-center datasets to enhance the scalability and reliability of ML models. Standardized reporting of sample size and its impact on model performance is essential for advancing the transparency and reproducibility of ML-based CVD risk prediction.

## 6. Practical Implications and Challenges of Machine Learning-Based Risk Prediction Models

The development and application of ML-based models in CVD risk prediction raise several important questions about their practical implications in clinical management. One key consideration is how improved prediction capabilities will translate into actionable interventions. Traditional, clinically based prediction models rely on modifiable risk factors, such as blood pressure, cholesterol levels, and smoking status, allowing for clinicians to target these factors for treatment and behavioral modifications. In contrast, ML-based models may identify predictors such as genetic factors, race, or socioeconomic variables that are not directly modifiable through current interventions. To address this challenge, future research and clinical strategies will need to focus on integrating ML-based insights into actionable care pathways. For instance, while genetic predispositions cannot be altered, their identification could lead to earlier screening and more personalized preventive measures. Similarly, socio-environmental predictors could inform public health initiatives aimed at addressing systemic inequalities that contribute to cardiovascular risk. Another important consideration is whether a universal ML-based model can be effectively applied across diverse populations or whether region-specific models will be necessary. Variations in genetic, environmental, and healthcare factors between countries, regions, or even hospitals may limit the generalizability of a single model. Developing localized models tailored to specific populations may offer greater accuracy and clinical relevance, but this approach also presents challenges in terms of resource requirements, validation, and scalability. Finally, the integration of ML-based models into clinical workflows must balance predictive accuracy with interpretability and clinical utility. Tools such as explainable AI and frameworks for translating complex predictors into actionable insights will be critical for fostering clinician trust and facilitating the adoption of these models in everyday practice.

## 7. Future Directions and Recommendations

To reduce the global burden of CVD, prevention efforts should emphasize primordial and primary prevention strategies informed by robust ML models. Future research must prioritize the following:

- Scalability and Validation: Conduct large-scale studies with diverse and geographically representative populations to ensure external validity.
- Model Integration: Develop frameworks for integrating heterogeneous data types, including imaging, omics, and clinical records, into unified predictive models.
- Standardization: Implement standardized reporting guidelines and coding systems, such as TRIPOD and UMLS, respectively, to improve data quality and model generalizability.
- Transparency and Interpretability: Foster clinician trust through interpretable algorithms that clearly elucidate the rationale behind predictions.
- Impact Assessment: Evaluate the effectiveness, cost-effectiveness, and real-world clinical utility of ML models to inform healthcare policies and decision-making.

By addressing these challenges and focusing on a data-driven, standardized approach to ML development, validation, and implementation, researchers can unlock the full potential of AI in improving CVD risk prediction and management. Integrating novel biomarkers, such as lipidomic and proteomic data, alongside advanced computational methods offers a transformative opportunity to enhance precision medicine and deliver equitable cardiovascular care.

## 8. Conclusions

The integration of ML and AI into CVD risk prediction represents a significant advancement in precision medicine, offering unprecedented opportunities to enhance patient outcomes management through improved predictive accuracy, interpretability, and personalized risk stratification. By leveraging diverse data sources and capturing complex, nonlinear relationships, ML and AI models effectively address many of the inherent limitations of traditional risk prediction tools. However, the widespread clinical adoption of these technologies remains contingent upon resolving critical challenges related to generalizability, accessibility, and transparency. While AI algorithms have demonstrated substantial promise in transforming CVD risk assessment, the number of ML models successfully implemented in clinical practice remains limited. A key obstacle lies in defining clear criteria for determining the point at which these models provide meaningful clinical benefits, a gap that continues to hinder their real-world applicability. Additionally, the inherent complexity of ML-based risk prediction tools often translates into increased computational demands, time constraints, and higher costs, which can compromise their usability in resource-limited clinical settings. This necessitates a balanced approach that optimizes predictive accuracy while maintaining operational feasibility to ensure seamless integration into healthcare workflows.

To address these challenges, future research must focus on improving the discriminatory power of ML models to distinguish between patients with unique risk factor combinations, accurately stratifying absolute CVD risk levels. Contemporary and emerging risk factors, including social determinants of health, behavioral data, and environmental exposures, should be rigorously evaluated for their incremental value in risk prediction. Furthermore, the integration of high-dimensional data from multi-omics approaches—such as genomics, proteomics, lipidomics, and metabolomics—alongside advanced imaging and clinical parameters will be critical in enhancing the precision and dynamic nature of CVD risk models. Despite their complexity, ML models do not always outperform conventional approaches like logistic regression or clinician-led predictions in certain contexts, as highlighted in systematic reviews. This observation underscores the importance of adopting a measured and pragmatic perspective, recognizing that risk assessment is inherently probabilistic and subject to assumptions, data quality, and analytic frameworks. Addressing these limitations requires robust external validation of ML models across heterogeneous and geographically diverse populations to ensure reliability, reproducibility, and applicability. Moreover, current geographical imbalances, low reproducibility rates, insufficient reporting quality, and a lack of standardized assessment frameworks must be systematically overcome to enable the development of unbiased and equitable ML models.

## Figures and Tables

**Table 1:** Comparison of ML, DL, and traditional models for CVD risk prediction: a summary of key studies and performance metrics.

First Author and Publication Year	Modeling Methods			Best Performed Model (Based on Internal Validation)	Performance Measures	Values of the Importance Measures with 95% CI, If Any (For the Best-Fitted Model)
	Machine Learning Algorithms	Deep Learning Algorithms	Traditional Risk Models or Statistical Analysis Methods			
Wu et al., 2024 [21]	Basic model (developed using Ridge regression and FRS variables), LRS full model (developed using Ridge regression and residual adjusted FRS), simplified LRS model (developed using LASSO regression and residual adjusted FRS)	None	FRS	LRS full model	AUC value and NRI	In the intermediate risk group: AUC: 0.659, NRI (over FRS): 0.36
Dong et al., 2024 [23]	XGBoost	None	Standard Cox PH model, FRS, UKPDS and the Chinese ASCVD model	XGBoost	C-index	Male: C-index: 0.741 (0.734, 0.748). Female: C-index: 0.764 (0.758, 0.770)
Shen et al., 2024 [25]	RF, SVM, LightGBM, and XGBoost	MLP	LR	For events within 3 and 5 years: MLP. For events within 8 years: SVM	Accuracy, sensitivity, specificity, and AUC value	MLP for 3-year events: Accuracy: 0.765, Sensitivity: 0.765, Specificity: 0.765, AUC: 0.803. MLP for 5-year events: Accuracy: 0.646, Sensitivity: 0.641, Specificity: 0.646, AUC: 0.694. SVM for 8-year events: Accuracy: 0.722, Sensitivity: 0.717, Specificity: 0.723, AUC: 0.753.
Yu et al., 2024 [26]	None	Dynamic-DeepHit	PCE	PCE for black males, Dynamic-DeepHit for the whole sample, other * males, black females, and other females	AUC, Brier score, and NRI	For the whole sample: AUC: 0.815 (0.782–0.844), NRI (over PCE): 0.385, and Brier Score: 0.051. AUC for Other males: 0.801 (0.753–0.848); Other females: 0.801 (0.737–0.864); Black males: 0.826 (0.756–0.897); Black females: 0.821 (0.751–0.890).
Atehortúa et al., 2023 [22]	XGBoost (with FRS variables), XGBoost (with exposome data), Biological + Clinical model (AutoPrognosis), and XGBoost (full model with exposome, biological, and clinical data)	None	FRS	XGBoost (full model with exposome, biological, and clinical data)	Sensitivity, specificity, precision, and AUC value	For general prediction: Sensitivity: 0.72, Specificity: 0.76, Precision: 0.75, AUC: 0.82. For 5-year prediction: Sensitivity: 0.73, Specificity: 0.76, Precision: 0.75, AUC: 0.83. For 9-year prediction: Sensitivity: 0.70, Specificity: 0.77, Precision: 0.75, AUC: 0.81. For 13-year prediction: Sensitivity: 0.74, Specificity: 0.79, Precision: 0.77, AUC: 0.82.
Lip et al., 2023 [27]	None	MLP	LR	MLP and LR (similar performance)	C-index	C-index: 0.68
Yoshida et al., 2023 [28]	Elnet-Cox, XGBoost, and Ensemble (average predictions of the Elnet-Cox and XGBoost)	None	Standard Cox PH model	Standard Cox PH model	C-index	For complete case data: C-index: 0.739. For imputed data: C-index: 0.748.
Deng et al., 2023 [29]	None	Deepsurv, Cox-nnet, and Nnet-survival	PCE and Cox PH-TWI model	PCE for black males, DeepSurv and Cox PH-TWI for white males (similar performance), DeepSurv for black females and white females	C-index	C-index for White males: 0.7371; White females: 0.797; Black males: 0.698; Black females: 0.789.
Lauber et al., 2022 [19]	Ridge regression	None	Standard Cox PH model	Ridge regression for N model (age+sex), L model (lipidomics), N+L+P model (age+sex+lipidomics+polygenic score), and N+L+P+C model (age+sex+lipidomics+genetics+standard clinical and vital measures). Standard Cox PH for P model (polygenic score)	AUC value	N model: AUC: 0.517, P model: AUC: 0.534, L model: AUC: 0.661, N+L+P: AUC: 0.634, and N+L+P+C: AUC: 0.659
Sajid, Almehmadi et al., 2021 [30]	Linear SVM, RBF SVM, and RF	ANN	LR	ANN (based on all performance measures except sensitivity) and Linear SVM (based on all performance measures)	Sensitivity, specificity, accuracy, AUC, Kappa-statistic, RMSE, and NRI	ANN (Sensitivity: 0.780, Specificity: 0.848, Accuracy: 0.8109, AUC: 0.871, Kappa-statistic: 0.622, RMSE: 0.378, and NRI (over LR): 3.7%). Linear SVM (Sensitivity: 0.809, Specificity: 0.809, Accuracy: 0.8086, AUC: 0.864, Kappa-statistic: 0.617, RMSE: 0.382, and NRI (over LR): 2.7%).
Sajid, Muhammad et al., 2021 [31]	RBF SVM, SGD-SVM, and RF	ANN	LR	RF (for both train-test split and cross-validation model approaches)	Sensitivity, specificity, precision, accuracy, and AUC	10-fold cross-validation approach: Sensitivity: 0.774, Specificity: 0.787, Precision: 0.785, Accuracy: 0.781, and AUC: 0.850. Train-test split approach: Sensitivity: 0.840, Specificity: 0.760, Precision: 0.804, Accuracy: 0.801, and AUC: 0.853.
Hoogeveen et al., 2020 [20]	XGBoost (with clinical data), XGBoost (with protein data), and XGBoost (with clinical and protein data)	None	None	XGBoost (with clinical and protein data)	AUC value	For 20-year MI risk prediction: AUC: 0.764 (0.749, 0.779). For 3-year MI risk prediction: AUC: 0.808.
An et al., 2021 [24]	SVMsmo, RF, and LightGBM	Bi-LSTM, A-LSTM, DeepRisk (based on A-LSTM), Deepr, Dipole, and R-MeHPAN	LR	DeepRisk (based on A-LSTM)	Precision, recall, F1-score, and AUC value	DeepRisk without demographics (Recall: 0.8056, Precision: 0.6717, F1-Score: 0.7325, AUC: 0.8219). DeepRisk with demographics (Recall: 0.8149, Precision: 0.6740, F1-Score: 0.7378, AUC: 0.8375).

Abbreviations: ASCVD: Atherosclerotic Cardiovascular Disease; AUC: Area Under the Curve; A-LSTM: Attention-based Bidirectional Long-Short Term Memory; Bi-LSTM: Bidirectional Long-Short Term Memory; CI: Confidence Interval; Cox PH: Cox Proportional Hazards; Cox PH-TWI: Two-Way Interactions Cox Proportional Hazards model; CVD: Cardiovascular Disease; DL: Deep Learning; Elnet-Cox: Elastic-net regularized Cox; FRS: Framingham Risk Score; LASSO: Least Absolute Shrinkage and Selection Operator; LightGBM: Light Gradient Boosting Machine; Linear SVM: Linear Support Vector Machine; LR: Logistic Regression; LRS: Lipidomic-enhanced Risk Score; MI: Myocardial Infarction; ML: Machine Learning; MLP: Multilayer Perceptron; NRI: Net Reclassification Improvement; PCE: Pooled Cohort Equations; RBF SVM: Radial Basis Function Support Vector Machine; RF: Random Forest; RMSE: Root Mean Square Error; SGD-SVM: Stochastic Gradient Descent based Support Vector Machine; SVM: Support Vector Machine; SVMsmo: Support Vector Machines with Sequential Minimal Optimization; UKPDS: UK Prospective Diabetes Study; and XGBoost: EXtreme Gradient Boosting. * Other females indicate White, Hispanic, and Asian females.

**Table 2:** Overview of predictors, validation methods, and endpoints in ML, DL, and traditional models for CVD risk prediction.

First Author and Publication Year	Final Predictors		Stratification Based on Gender and/or Race	Model Validation		Length of Follow-Up	Endpoint of Interest
	Methods to Select Final Predictors [and Number of Predictors Only for the Best-Performed Model]	Type of Predictors		**Internal**	**External**		
Wu et al., 2024 [21]	For basic model and LRS full model: No method for feature selection. For simplified LRS model: LASSO regression [705 lipids + residual adjusted FRS]	Lipidomic data and traditional FRS risk factors	No	10-fold cross-validation	Yes	Median (IQR) in year: 5.1 (2.5–7.9)	10-year CVD risk prediction
Dong et al., 2024 [23]	For ML models: Boruta method. For the 2nd ML model: Expert opinion for exclusion of doubtful features. For Cox PH models: Backward stepwise selection [For 2nd ML models: 13 features for female-specific model and 12 features for male-specific model]	Demographic attributes, disease characteristics, clinical factors, and treatment modalities	Yes (gender)	Train-test split	No	Median in year: 9.75	10-year CVD risk prediction
Shen et al., 2024 [25]	No method for feature selection but expert opinion and features with cases number ≤ 10 or low correlation coefficients with the outcome were excluded [8 features]	Demographic attributes, comorbid history, and clinical factors	No	Train-test split	No	Maximum: 8-year	Stroke risk prediction
Yu et al., 2024 [26]	No method for feature selection [10 features]	Longitudinal data [demographic attributes, comorbid history, clinical and behavioural factors (some of them are PCE factors)]	Yes (gender and race)	Train-tune-test split	No	10-year	10-year ASCVD risk prediction
Atehortúa et al., 2023 [22]	No method for feature selection [156 features]	Exposome (physical measures, environmental, lifestyle, traumatic and psychosocial events, sociodemographic and early-life factors), biological and clinical factors	No	Nested cross-validation	Yes	Maximum: 13-year	CVD risk prediction
Lip et al., 2023 [27]	No method for feature selection [22 features]	Comorbid history, lifestyle/personal factors, healthcare utilization history, and demographic attributes	No	Train-test split	No	Maximum: 45.2 months	CVD risk prediction
Yoshida et al., 2023 [28]	No method for feature selection but features with an absolute correlation ≥ 0.8 were removed [35 features]	Demographic attributes, laboratory tests, and questionnaire features	No	Train-validation-test split	No	Median in year: 4	Stroke risk prediction
Deng et al., 2023 [29]	No method for feature selection [7 features]	Traditional PCE risk factors	Yes (gender and race)	10 × 10 cross-validation	Yes	Mean (SD) in year: 10.50 (3.02)	ASCVD risk prediction
Lauber et al., 2022 [19]	No method for feature selection [2 demographic features + 184 lipid species + 1 polygenic score + 7 clinical features]	Lipidomic data, genotype data, standard clinical and vital measurements	No	10-fold cross-validation	No	Maximum: 23-year	CVD risk prediction
Sajid, Almehmadi et al., 2021 [30]	Bivariate odds ratio analysis [14 features]	Nonlaboratory features (demographic attributes, comorbid history, parental CVD history, behavioral factors, physical activity, and dietary features)	No	Train-test split and 10-fold cross-validation (for ML- and deep learning-based models)	No	Without follow-up (case-control study)	CVD risk prediction
Sajid, Muhammad et al., 2021 [31]	No method for feature selection [8 features]	Nonclinical features	No	Train-test split and 10-fold cross-validation	No	Without follow-up (case-control study)	CVD risk prediction
Hoogeveen et al., 2020 [20]	Proteins were excluded if ≥90% of the values were below lower limit of detection. Selection by relative importance of a panel of 50 proteins (the proteins in the panel are different for 3- and 20-year prediction) [50 features]. No method for clinical features selection [10 features]	Protein and clinical factors (traditional risk factors from FRS, PCE, and SCORE)	No	Train-test split	Yes	Median in year: 20	20-year MI risk prediction and 3-year MI risk prediction
An et al., 2021 [24]	No method for feature selection but laboratory indexes with low frequency (occurring less than 100 times) and/or missing values ratio exceeding 0.9 were removed [817 diagnosis codes + 687 laboratory indexes + 5 demographics]	Diagnosis codes, laboratory features, and demographic features	No	Train-validation-test split	Νο	1-year	CVD risk prediction

Abbreviations: ASCVD: Atherosclerotic Cardiovascular Disease; Cox PH: Cox Proportional Hazards; CVD: Cardiovascular Disease; DL: Deep Learning; FRS: Framingham Risk Score; IQR: Interquartile Range; LASSO: Least Absolute Shrinkage and Selection Operator; LRS: Lipidomic-enhanced Risk Score; MI: Myocardial Infarction; ML: Machine Learning; and SCORE: Systemic Coronary Risk Evaluation.

**Table 3:** Characteristics of study populations, regions, and study designs in CVD risk prediction research.

First Author and Publication Year	Population	Study Region	Setting	Sample Size	Age	Gender (% Female)	Study Type
Wu et al., 2024 [21]	Individuals without a history of CVD	Australia	Community [Australian Diabetes, Obesity, and Lifestyle Study (AusDiab)]	7895 individuals. For external validation, 3988 individuals.	Mean age (SD) in year: 50.5 (13.4). In the external validation cohort, mean age (SD) in year: 49.8 (16.8).	57.1%. In the external validation cohort, 57.4%.	Prospective
Dong et al., 2024 [23]	T2DM patients aged 18 years and older without a history of CVD	China	Institution (Clinical Management System of the Hong Kong Hospital Authority)	141,516 individuals (female: 78,078, male: 63,438)	(Model Development) Male: mean age (SD) in year: 63.09 (11.45). (Model Validation) Male: mean age (SD) in year: 63.07 (11.45). (Model Development) Female: mean age (SD) in year: 65.58 (11.87). (Model Validation) Female: mean age (SD) in year: 65.71 (11.78).	55.2%	Retrospective
Shen et al., 2024 [25]	Patients aged 20 years and older with LUTS but without a history of CVD	Taiwan	Institution (Chi Mei Medical Center hospital information system database)	1799 individuals	Mean age (SD) in year: 59.4 (14.8)	100%	Retrospective
Yu et al., 2024 [26]	Participants aged 40–75 years without a history of ASCVD	US	Community [the Framingham Heart Study, Framingham Offspring Study, Coronary Artery Risk Development in Young Adults (CARDIA) Study, and Atherosclerosis Risk in Communities (ARIC) Study]	15,565 individuals	Mean age (SD) in year: 50.2 (7.2)	54.8%	Prospective
Atehortúa et al., 2023 [22]	Participants aged 37–73 years without a history of CVD	Europe	Institution [UK Biobank (UKBB)]	9658 individuals (4829 participants per class, i.e., control group and individuals at risk of CVD). For external validation, 1038 individuals (519 participants per class).	Mean age (SD) in year for CVD class 74.9 (6.6) and for control class without CVD 69.9 (8.0). In the external validation cohort, mean age (SD) in year for CVD class 74.8 (7.2) and for control class without CVD 70.0 (8.5).	34.3% for CVD class and 56.7% for control class without CVD. In the external validation cohort, 35.8% for CVD class and 56.2% for control class without CVD.	Prospective
Lip et al., 2023 [27]	Individuals aged 65 years and older, including those with some level of disability aged 18 years and older, in the absence of prior CVD and the presence of non-cardiovascular multi-morbidity	US	Institution (Medicare health plan)	154,551 individuals	Mean age in year: 68.8	62.2%	Retrospective
Yoshida et al., 2023 [28]	Individuals aged 30–74 years without a history of IHD, stroke, and/or cancer	Japan	Institution (IQVIA Claims Database)	572,971 individuals	Mean age (SD) in year: 46.46 (9.45)	46.0%	Retrospective
Deng et al., 2023 [29]	Individuals aged 40–75 years who are free of ASCVD and a previous history of myocardial infarction, stroke, congestive heart failure, or atrial fibrillation	US	Community [Lifetime Risk Pooling Project, including Atherosclerosis Risk in Communities (ARIC) study, Cardiovascular Health Study (CHS), Framingham Offsprinig study, Coronary Artery Risk Development in Young Adults (CARDIA) study, the Framingham Original study]	23,216 individuals. For external validation, 4259 individuals.	Mean age (SD) in year: 57.8 (9.6). In the external validation cohort, mean age (SD) in year: 61.6 (9.6).	56.93% (both white and black). In the external validation cohort, 53.2% (both white and black).	Prospective
Lauber et al., 2022 [19]	Individuals without a history of CVD	Europe	Community [Malmö Diet and Cancer-Cardiovascular Study (MDC-CC)]	3951 individuals	NR	NR	Prospective
Sajid, Almehmadi et al., 2021 [30]	Participants aged 30–76 years without a history of CVD	Pakistan	Institution [Punjab Institute of Cardiology (PIC)]	460 individuals (230 cases and 230 controls)	Mean age (SD) in year: 48.0 (11.31)	32.2%	Retrospective
Sajid, Muhammad et al., 2021 [31]	Participants aged 30 years and older without a history of CVD	Pakistan	Institution [Punjab Institute of Cardiology (PIC)]	460 individuals (230 cases and 230 controls)	Mean age in year: 48.0	32.2%	Retrospective
Hoogeveen et al., 2020 [20]	Participants aged 39–79 years without a history of CVD	Europe	Institution [European Prospective Investigation (EPIC)-Norfolk study]	822 individuals (411 cases and 411 controls). For external validation, 702 individuals.	Sample of EPIC-case: mean age (SD) in year: 66 (7.8). Sample of EPIC-control: mean age (SD) in year: 62 (7.7). In the external validation for the sample of cases: mean age (SD) in year: 55 (8.1). In the external validation for the sample of controls: mean age (SD) in year: 54 (8.2).	Sample of EPIC-case: 31.4%. Sample of EPIC-control: 38.2%. In the external validation for the sample of cases: 66.7%. In the external validation for the sample of controls: 67%.	Prospective
An et al., 2021 [24]	Patients with diabetes, hypertension, or hyperlipidemia but without a history of CVD	China	Institution (Xiangya Medical Dataset)	146,296 individuals	Mean age in year: 45.07	NR	Retrospective

Abbreviations: ASCVD: Atherosclerotic Cardiovascular Disease; CVD: Cardiovascular Disease; IHD: Ischemic Heart Disease; LUTS: Lower Urinary Tract Symptoms; NR: Not Reported; SD: Standard Deviation; T2DM: Type 2 Diabetes Mellitus; and US: United States.

## Data Availability

Not applicable.

## Abbreviations

The following abbreviations are used in this manuscript:

AI	Artificial Intelligence
ANN	Artificial Neural Networks
ARIC	Atherosclerosis Risk in Communities
ASCVD	Atherosclerotic Cardiovascular Disease
AUC	Area Under the Curve
AUROC	Area Under the Receiver Operating Characteristic curve
AusDiab	Australian Diabetes, Obesity, and Lifestyle Study
A-LSTM	Attention-based Bidirectional Long-Short Term Memory
Bi-LSTM	Bidirectional Long-Short Term Memory
BMI	Body Mass Index
CAC	Coronary Artery Calcification
CARDIA	Coronary Artery Risk Development in Young Adults
CCTA	Coronary Computed Tomography Angiography
CHS	Cardiovascular Health Study
CI	Confidence interval
cIMT	Carotid Intima-Media Thickness
CMR	Cardiac Magnetic Resonance
Cox PH	Cox Proportional Hazards
Cox PH-TWI	Two-Way Interactions Cox Proportional Hazards
CT	Computed Tomography
CUS	Carotid Ultrasound
CVDs	Cardiovascular Diseases
C-index	Concordance index
DAGs	Diacylglycerides
DL	Deep Learning
ECG	Electrocardiographic
EHRs	Electronic Health Records
Elnet-Cox	Elastic-net Cox
EPIC	European Prospective Investigation
FRS	Framingham Risk Score
HbA1c	Glycated Hemoglobin
IHD	Ischemic Heart Disease
IQR	Interquartile Range
LASSO	Least Absolute Shrinkage and Selection Operator
LD	Lumen Diameter
LightGBM	Light Gradient Boosting Machine
Linear SVM	Linear Support Vector Machine
LR	Logistic Regression
LRS	Lipidomic Risk Score
LUTS	Lower Urinary Tract Symptoms
MACCE	Major Adverse Cardiac and Cerebrovascular Events
MACE	Major Adverse Cardiovascular Events
MAE	Major Adverse Events
MDC-CC	Malmö Diet and Cancer-Cardiovascular Study
MI	Myocardial infarction
MINIMAR	MINimum Information for Medical AI Reporting
ML	Machine Learning
MLP	Multilayer Perceptron
MRI	Magnetic Resonance Imaging
NACE	Net Adverse Clinical Events
NLP	Natural Language Processing
NR	Not Reported
NRI	Net Reclassification Improvement
PCE	Pooled Cohort Equations
PIC	Punjab Institute of Cardiology
PICOS	Patient, Intervention, Comparison, Outcomes, and Study
RBF SVM	Radial Basis Function Support Vector Machine
RF	Random Forest
RMSE	Root Mean Square Error
SBP	Systolic Blood Pressure
SCORE	Systematic Coronary Risk Evaluation
SD	Standard Deviation
SGD-SVM	Stochastic Gradient Descent based Support Vector Machine
SHAP	SHapley Additive exPlanations
SVM	Support Vector Machine
SVMsmo	Support Vector Machines with Sequential Minimal Optimization
TAGs	Triacylglycerides
TRIPOD	Transparent Reporting of a multivariable prediction model for Individual Prognosis Or Diagnosis
T2DM	Type 2 Diabetes Mellitus
UKBB	United Kingdom Biobank
UKPDS	United Kingdom Prospective Diabetes Study
UMLS	Unified Medical Language System
US	United States
XGBoost	EXtreme Gradient Boosting
